# A longitudinal study examining the effects of a season of American football on lipids and lipoproteins

**DOI:** 10.1186/s12944-015-0021-6

**Published:** 2015-04-21

**Authors:** Jonathan M Oliver, Dustin P Joubert, Aaron Caldwell, Steve E Martin, Stephen F Crouse

**Affiliations:** Department of Kinesiology, Texas Christian University, TCU Box 297730, Fort Worth, TX 76129 USA; Department of Health and Kinesiology, Texas A&M University, 4245 TAMU, College Station, TX 77843 USA

**Keywords:** Obesity, Cardiovascular disease risk, Correlation, Athletes

## Abstract

**Background:**

Dyslipidemia is one factor cited for increased risk of cardiovascular disease (CVD) in American football players. However, American football players undergo physical conditioning which is known to influence lipids. This study examined if the physical activity of an American football season is associated with changes in lipids and if a relationship exists between lipids and body composition.

**Methods:**

Fourteen division I freshmen American football players had blood drawn prior to summer training (T1), end of competition (T2), and end of spring training (T3). Samples were analyzed for total cholesterol (TCHL), HDL-C, LDL-C, and triglycerides (TG). Body composition was assessed via dual-x-ray absorptiometry. National Cholesterol Education Program (NCEP) lipid categorization was used to characterize participants. Pearson correlations were computed to determine relationships.

**Results:**

Body mass increased T2 (p = 0.008) as a result of increase in fat mass (p = 0.005) and remained high despite a decrease T3. Lean mass did not differ significantly at any time. No significant time effects were observed for lipids measured. The number of participants presenting with risk factors attributed to dyslipidemia varied. By T3, no participant was categorized as “low” for HDL-C. TCHL was moderately correlated (r = 0.60) with fat mass at T1; whereas a moderate correlation (r = −0.57) was observed between BMI and HDL-C at T2. TG was strongly correlated with fat mass at each time point (T1, r = 0.83; T2, r = 0.94; T3, r = 0.70).

**Conclusion:**

The physical activity associated with a season of football results in little change in blood lipids and CVD risk. Further, TG are strongly related to fat mass. Future research should focus on examining the cause of dyslipidemia in American football players.

## Introduction

American football athletes undergo intense physical training, often exceeding the energy expenditure requirements generally recognized as effective in altering lipids and lipoproteins in a single work out [1]. However, despite this level of physical activity and the purported beneficial effects of physical activity on lipids and lipoproteins [1], a number of studies have reported American football players have an increased risk for cardiovascular disease (CVD) attributed to dyslipidemia [2-4]. While often thought to only affect those in positions associated with larger size, data from seven different high schools and one National Collegiate Association (NCAA) Division I American football team showed that dyslipidemia is not limited to only those larger individuals, as 47.1% of those players categorized as “Athletic” and 28.6% identifying as “Skill” presented with high-density lipoprotein cholesterol (HDL-C) concentrations associated with an increased risk of CVD [5]. Even more concerning, given the continued increase in youth participation in the sport of American football [6], 54.3% of the high school players tested had an increased risk of CVD due to low HDL-C and 13.0% had elevated triglycerides (TG) [5]. Thus, there exists a need for further study into the potential effects that the physical activity associated with the sport of American football has on risk factors associated with CVD attributed to dyslipidemia.

Previous studies in American football athletes have been limited to cross-sectional data [2-5,7] or serial data resulting from interventions not necessarily indicative of the physical activity associated with a traditional season [8]. Given the data regarding the beneficial effects of physical activity on lipids and lipoproteins [1], the relationship between dyslipidemia and CVD [9,10], and the recent reports of greater CVD risk attributed to dyslipidemia among American football players of all ages [2,4,5]; our goal was to examine changes in blood lipid and lipoproteins over the course of a traditional American football season, as physical activity and nutritional supervision are known to differ across this time period. We hypothesized that a more favorable lipid profile would be present in athletes as intensity increased over the course of the season. The relationship between body composition and blood lipids was also investigated as others have reported on this relationship and its role in CVD risk [4,7]. Thus, we further hypothesized a relationship would exist between measures of body composition, specifically those related to body mass and fat mass, and blood lipids.

## Methods

### Experimental approach to problem

To determine the effect of a season of American football to include competition and training on serum lipid and lipoprotein particle numbers in freshmen athletes a longitudinal research design was employed. Blood samples were obtained at three distinct time-points over the course of an athletic year corresponding with changes in physical activity, nutritional supervision, and competition associated with a season of American football. An initial sample was obtained prior to participation in any collegiate training and/or competition (Pre-Season). Approximately 7 months following the initial sample, corresponding with the end of the competitive season, a secondary sample was collected (Post-Competition). A third sample was obtained approximately 5 months later at the conclusion of spring practice (Post-Season), corresponding with the end of the academic year.

### Subjects

Fourteen (n = 14) freshmen American football athletes entering their first year of competition at an NCAA Division I university volunteered to participate in this study. All participants were deemed healthy for athletic competition by a team physician, thus no exclusionary criteria were used. This study was conducted according to the Declaration of Helsinki guidelines. All procedures involving human subjects were approved by the Institutional Review Board of Texas A&M University for use of human subjects. Written consent was obtained from all participants.

### Demographic determination

Height and body mass were recorded to the nearest .01 cm and .02 kg, respectively, using a Detecto stadiometer and scale (Webb City, MO) with participants in socks or bare feet. Body composition was subsequently determined in a sub sample (n = 7) using dual x-ray absorptiometry (DXA) (Lunar Prodigy, General Electric; Madison, WI) calibrated according to manufacturer’s guidelines and performed by a trained technician. Previous studies indicate DXA to be an accurate and reliable means to assess changes in body composition [11]. The percent coefficient of variation as assessed by the performance of repeated calibrations (10 days) for the equipment used in this study was 0.11% and 0.11% for lean and fat, respectively.

### Blood collection

The night prior to each blood collection, subjects were verbally reminded to ingest only water after 2200. On the day of blood sampling, subjects reported to the laboratory after an overnight fast (~10 hours), verbally verified prior to collection. After being seated in a phlebotomy chair, blood was collected via venipuncture from the antecubital fossa region into two separate serum separator vacutainer tubes using standard, sterile phlebotomy procedures. To prevent any substantial clinical differences in subsequent blood draws, short-term stasis was prevented by immediate removal of the tourniquet following observable blood flow [12]. After collection, blood was allowed to clot at room temperature and was then centrifuged in a refrigerated centrifuge for 20 minutes (2,000 × G) for serum separation. One serum separator vacutainer tube was couriered the same day to another laboratory for determination of total cholesterol (TCHL), HDL-C, low-density lipoprotein cholesterol (LDL-C) and TG. Aliquots of serum from the second tube were then transferred into separate labeled tubes and frozen at −80°C until further analysis for lipoprotein particles.

### Physical activity

The period between the first two collections (Pre-Season and Post-Competition) included vigorous two-a-day practices as well as the competitive season. Two-a-day practices consisted of a high volume of anaerobic conditioning, skills, drills, and strength training. During the competitive season, practice was held approximately three times per week in which subjects again participated in heavy anaerobic conditioning, drills, and skills training. Strength training was also performed twice per week. Dietary supervision during this time followed guidelines set forth by the NCAA. Recovery meals were provided in the form of a training table following practice sessions. Additionally, lunch and dinner were provided twice per week. Game day nutrition included pre-game and post-game meals with additional training table provided during competition.

Following Post-Competition, but preceding Post-Season, subjects underwent winter conditioning which included vigorous anaerobic conditioning, lasting approximately one month. Spring practice began approximately 2 months prior to the Post-Season blood draw. Practices included anaerobic conditioning, skills, and drills performed three times per week. Strength training was performed at least twice per week. Dietary supervision included snacks provided on a training table post-activity. Additionally, meals were provided four times a week, equally distributed between lunch and dinner.

Meals, snacks, and training tables were provided in accordance with the NCAA rules and regulations only. No attempt was made to control for dietary intake. Further, no dietary data were recorded for the duration of the study.

### Lipid determination

TCHL, HDL-C and TG were measured using commercially available enzymatic kits (Abbott Laboratories; Abbott Park, IL) on an Abbott Architect ci8200 (Abbott Diagnostics; Lake Forest, IL) by a Clinical Laboratory Improvement Amendments (CLIA) certified laboratory. Enzymatic, accelerator selective detergent and glycerol phosphate oxidase were the methods of determination for TCHL, HDL-C and TG respectively. LDL-C concentrations were calculated using the Friedewald equation [13].

### Lipoprotein particle number determination

Lipoprotein particle numbers were determined using the lipoprotein subgroup particle number analysis method (Spectracell Laboratories; Houston, TX). This lipoprotein particle separation procedure utilizes a patented method (Patent No.: US 7,856,323 B2) with a continuous gradient generated by analytical ultracentrifugation. The lipoprotein particles were stained with a fluorescent dye and then separated in the gradient over a range of d = 1.000 - 1.300 g*cm-3. After separation, the fluorescence of the lipoprotein particles was measured in an HPLC-type flow system and normalized to a cholesterol scale with a proprietary algorithm. Values for each lipoprotein subgroup at their specific densities were determined using a multiple Gaussian fit/integration routine. The total number of very low-density lipoprotein (VLDL), low-density lipoprotein (LDL-T), remnant lipoprotein (RLP), dense low-density lipoprotein III (Dense LDL III), dense low-density lipoprotein IV(Dense LDL IV), high-density lipoprotein (HDL-T), and buoyant high-density lipoprotein 2b (Buoyant HDL 2b) particles were determined at their specific densities. The coefficient of variation for this analysis using known standards has been reported as 2-3%. The mean size was determined by calculating the mean particle size from the weighted LDL subgroups using the size/density relationship.

### Statistical analyses

All statistical analyses were performed using IBM SPSS V21.0 (Chicago, IL). A one-way repeated measures analysis of variance (ANOVA) was used to compare changes over time in variables of interest. Comparisonwise error rate was set at P ≤ 0.05. Trends were identified if a P value was ≤ 0.10 but > 0.05. Least Square Difference (LSD) post hoc tests were used when a significant finding or trend was observed. The National Cholesterol Education Program Adult Treatment Panel III lipid categorization [14] was used to determine the number of participants within the desired range. Bivariate (Pearson) correlations were computed to determine significant relationships between fat mass and body mass index (BMI) at each time point with corresponding blood lipid value. Moderate correlations were defined as r values of 0.41 to 0.70 and strong correlations were considered when values were between 0.71 and 0.90. Significance was set at P ≤ 0.05. All data are presented as mean ± standard deviation (SD) unless otherwise noted.

## Results

All participants were 18 years of age. Participants were from the following positions: defensive line (n = 2), offensive line (n = 2), defensive back (n = 3), kicker (n = 2), linebacker (n = 3), quarterback (n = 1), and wide receiver (n =1). Changes in body composition over the course of the season are presented in Table 1. The increase in body mass Post-Competition (p = 0.008) resulted from an increase in fat mass (2.98 ± 1.79 kg; p = 0.005). However, body mass remained stable Post-Competition despite a small decrease in fat mass with no statistically significant difference observed in lean mass.

**Table 1:** **Change in body composition over the course of the season**

	**Pre-Season**	**Post-Competition**	**Post-Season**
Body mass (kg)	95.21 ± 18.31	98.74 ± 17.85^†^	98.81 ± 15.71^†^
Fat mass (kg)*	13.96 ± 10.86	16.94 ± 11.59^†^	14.95 ± 9.61¤
Lean mass (kg)*	81.29 ± 12.69	82.14 ± 8.96	83.60 ± 8.49
BMI	26.9 ± 4.2	27.9 ± 4.1^†^	27.9 ± 3.5^†^

Data are mean ± SD. ^†^Significantly different from Pre-Season value (p < 0.05). ¤Significantly different from Post-Competition value (p < 0.10). *Only 7 participants included in analysis.

Blood lipids for each of the three time-points with their corresponding 95% confidence interval are presented in Table 2. There were no main effects for time in any of the lipids measured.

**Table 2:** **Blood lipids over the course of the season**

	**Pre-Season**	**Post-Competition**	**Post-Season**
TCHL (mmol•L^−1^)	4.2 (3.7 – 4.7)	4.2 (3.8 – 4.7)	4.3 (3.8 – 4.8)
LDL-C (mmol•L^−1^)	2.5 (2.2 – 2.9)	2.6 (2.2 – 3.0)	2.5 (2.2 – 2.9)
HDL-C (mmol•L^−1^)	1.2 (1.1 – 1.4)	1.2 (1.1 – 1.4)	1.3 (1.2 –1.4)
TCHL:HDL-C ratio	3.45 (3.06 – 3.85)	3.58 (3.16 – 4.01)	3.33 (2.98 – 3.68)
TG (mmol•L^−1^)	0.9 (0.7 – 1.2)	0.9 (0.7 – 1.1)	1.1 (0.8 – 1.3)

Data are mean (95% CI). TCHL, Total cholesterol; LDL-C, low-density lipoprotein cholesterol; HDL-C, high-density lipoprotein cholesterol; TG, triglycerides.

The number of participants in each lipid category according to the NCEP ATP III classification guidelines is presented in Table 3. The highest category achieved in any lipid measure was “borderline high”. In terms of TCHL, by Post-Season 3 participants were in the “borderline high” category. This was the greatest number of participants in the “borderline high” category of any of the lipids categorized. By Post-Season no participant was categorized as “low” for HDL-C. The number of participants presenting with risk factors attributed to dyslipidemia was five (n = 5) Pre-Season, six (n = 6) Post-Competitions, and three (n = 3) Post-Season.

**Table 3:** **Number of participants in each lipid category defined by National Cholesterol Education Program**

**Pre-Season**	**Post-Competition**	**Post-Season**	
			Total Cholesterol
12	13	11	<5.2 (mmol•L^−1^) “Desirable”
2	1	3	5.2 – 6.2 (mmol•L^−1^) “Borderline high”
0	0	0	≥6.2 (mmol•L^−1^) “High”
			LDL-C
6	6	6	<2.6 (mmol•L^−1^) “Optimal”
6	7	7	2.6 – 3.3 (mmol•L^−1^) “Near optimal”
2	1	1	3.4 – 4.1 (mmol•L^−1^) “Borderline high”
0	0	0	4.1 – 4.9 (mmol•L^−1^) “High”
0	0	0	≥4.9 (mmol•L^−1^) “Very high”
			HDL-C
2	4	0	<1.0 (mmol•L^−1^) “Low”
10	9	12	1.0 – 1.5
2	1	2	≥1.6 (mmol•L^−1^) “High”
			Triglycerides
14	13	13	<1.7 (mmol•L^−1^) “Normal”
0	1	1	1.7 – 2.2 (mmol•L^−1^) “Borderline high”
0	0	0	2.3 – 5.6 (mmol•L^−1^) “High”

LDL-C, low-density lipoprotein cholesterol; HDL-C, high-density lipoprotein cholesterol.

TCHL and LDL-C were moderately correlated with fat mass but only at Pre-Season (Table 4), whereas a negative correlation was observed between BMI and HDL-C Post-Competition. A moderate correlation was observed Pre-Season between LDL-C and fat mass which approached significance. The greatest number and strongest correlations occurred between fat mass and TG and BMI and TG. TG were correlated with fat mass at each time point; whereas, the correlation between TG and BMI only occurred Pre-Season and Post-Competition.

**Table 4:** **Bivariate correlations between fat mass, BMI and lipid over the course of the season**

		**Fat Mass Pre-Season**	**Fat Mass Post-Competition**	**Fat Mass Post-Season**	**BMI Pre-Season**	**BMI Post-Competition**	**BMI Post-Season**
Total Cholesterol Pre-Season	r	0.604			0.432		
	P	0.049*			0.123		
	n	11			14		
Total Cholesterol Post-Competition	r		0.131			0.129	
	P		0.736			0.605	
	n		9			13	
Total Cholesterol Post-Season	r			0.390			0.283
	P			0.387			0.349
	n			7			13
LDL-C Pre-Season	r	0.528			0.348		
	P	0.095^†^			0.223		
	n	11			14		
LDL-C Post-Competition	r		0.005			0.099	
	P		0.990			0.748	
	n		9			13	
LDL-C Post-Season	r			0.361			0.351
	P			0.426			0.240
	n			7			13
HDL-C Pre-Season	r	0.329			0.209		
	P	0.323			0.474		
	n	11			14		
HDL-C Post-Competition	r		−0.536			−0.566	
	P		0.137			0.044*	
	n		9			0.13	
HDL-C Post-Season	r			−0.233			−0.425
	P			0.614			0.148
	n			7			13
Triglycerides Pre-Season	r	0.833			0.752		
	P	0.001*			0.002*		
	n	11			14		
Triglycerides Post-Competition	r		0.942			0.846	
	P		0.000*			0.000*	
	n		9			13	
Triglycerides Post-Season	r			0.702			0.442
	P			0.079^†^			0.130
	n			7			13

r represents Pearson correlations; P values represent 2-tailed testing; n represents sample size for bivariate correlation; *Significant at p ≤ 0.05; ^†^Significant at p ≤ 0.10 but > 0.05.

Changes in lipoprotein particle number are presented in Table 5. No main effect for time was observed in VLDL, LDL-T, Dense LDL III, Dense LDL IV, or buoyant HDL 2b. A non-significant trend (p = 0.062) was observed in LDL-T. Post-Competition LDL-T was significantly lower than Pre-Season (p = 0.023). A significant time effect was observed in RLP (p = 0.011). Similar to the trend observed in LDL-T, RLP decreased Post-Competition (−24.1 ± 21.0%; p = 0.006), but returned to baseline levels Post-Season. A non-significant trend (p = 0.063) was also noted in Dense LDL III which was significant when evaluated as percent change from baseline (p = 0.041). The Post-Season decrease (−9.4 ± 10.4%) was significantly different than both time-points (p < 0.05). Albeit small, the changes in LDL mean size over the course of the season were significant (p < 0.001) with a decrease noted Post-Competition (0.50 ± 0.61%; p = 0.010) and slight increase observed Post-Season (0.33 ± 0.35%; p = 0.003) when compared to Pre-Season.

**Table 5:** **Lipoprotein particle numbers over the course of the season**

	**Pre-Season**	**Post-Competition**	**Post-Season**
VLDL (nmol•L^−1^)	37 ± 11	37 ± 16	42 ± 24
LDL Total (nmol•L^−1^)	608 ± 163	546 ± 158^†^	594 ± 146¤
RLP (nmol•L^−1^)	94 ± 34	69 ± 25^†^	83 ± 33^‡^
Dense LDL III (nmol•L^−1^)	146 ± 52	159 ± 57	131 ± 46^†‡^
Dense LDL IV (nmol•L^−1^)	63 ± 13	65 ± 22	62 ± 13
LDL Mean Size (nm)	20.14 ± 0.12	20.04 ± 0.10^†^	20.21 ± 0.12^†‡^
HDL Total (nmol•L^−1^)	8916 ± 1719	9078 ± 1390	9123 ± 1642
Buoyant HDL 2b (nmol•L^−1^)	1495 ± 491	1314 ± 299	1388 ± 330

Data are mean ± SD. VLDL, very low-density lipoprotein; LDL, low-density lipoprotein; RLP, remnant lipoprotein; HDL, high-density lipoprotein; ^†^Significantly different from Pre-Season value (p ≤ 0.05). ^‡^Significantly different from Post-Competition (p ≤ 0.05). ¤Significantly different from Post-Competition value (p ≤ 0.10).

## Discussion

The purpose of this study was to examine the effect of a season of collegiate American football participation on serum lipid and lipoprotein particle numbers in freshmen athletes. A secondary purpose was to examine the relationship between blood lipids and body composition (fat mass and BMI) over the course of the season. To our knowledge this is the first study to investigate the effect of a traditional season of American football on changes in lipids and lipoproteins. Our results suggest that a season of American football is not associated with significant changes in traditionally measured lipid profiles of freshmen athletes. However, the number of participants presenting with risk factors as characterized by the NCEP did change over the course of the season. Further, TG was strongly correlated with fat mass, as well as BMI over the course of the season; while only moderate correlations were detected between blood lipids (TCHL, LDL-C, and HDL-C) and body composition, which were not consistent across the season. Few, characteristically positive, changes were reported in RLP and LDL mean size over the course of the season, but these were small.

The lack of significant change over the course of the season in LDL-C and TCHL is not unexpected as others have reported that more often than not, TCHL and LDL-C remain unaltered after exercise training [1]. However, the lack of change in HDL-C and TG suggest the need for further study as the energy expenditure from heavy physical training performed over the course of the season exceeds all previously suggested thresholds for significant adaptations in these two blood lipids. Further, Durstine et al. [1] concluded that changes in HDL-C as a result of exercise training are more effective when initial HDL-C is normal to high (≥37 mg•dL^−1^ or 0.96 mmol•L^−1^); while initial TG have little to do with changes following training. Those authors also concluded that decreased body fat mass is not necessary for changes to result. Given that only one participant Pre-Season and two Post-Competition presented with an HDL-C below the threshold suggested by Durstine et al. [1], one would expect significant increases in HDL-C. Interestingly, changes were noted in the number of participants presenting with risk factors for CVD according to the NCEP, and the greatest change occurred in HDL-C. By Post-Season no participant reported having an HDL-C categorized as “low”, in agreement with a trend observed in percent change from Pre-Season (7.5 ± 3.8%).

Still, the lack of change across the season in lipids requires further interpretation. Previous studies have reported on the relationship between dyslipidemia and obesity in this population of athletes [4,7]; however, those have been limited to cross sectional studies. To test this assertion across a season of training, we investigated the correlation between fat mass and BMI and lipid values. Only three moderate correlations were found at varying time points between fat mass or BMI and TCHL, LDL-C, and HDL-C. The lack of correlation between these measures and BMI is not surprising given we recently reported that the use of current BMI thresholds for obesity in American football athletes may result in misleading risk inferences about health [15]. We did observe strong correlations at all three time points between fat mass and TG. Borchers et al. [7] reported an association between percent body fat and TCHL, HDL-C, LDL-C, and TG in a cohort of Division I collegiate American football athletes. Similarly, Garry and McShane [16] demonstrated a direct relationship between BMI and TG, the TCHL/HDL-C ratio, and an inverse relationship between BMI and HDL-C. Our results are in contrast, given the lack of correlation between measures of obesity and TCHL, HDL-C, and LDL-C. However, the cross-sectional design, greater proportion of participants considered obese and presenting with an abnormal lipid profile may have contributed to these divergent findings. Despite recent evidence that the dyslipidemia is not limited to linemen [5], a larger number of players in those positions are obese and present with abnormal lipid profiles. Whether a similar relationship exists across a season in those athletes is unknown. Only four participants in the current study were classified as linemen.

In the current study, lipoprotein particle numbers were also examined over the course of the season. A debate exists regarding the clinical relevance of total LDL-C versus LDL size and sub-classification [17]. Recent research has suggested that LDL size is a very good predictor of a cardiovascular event in populations with an already high risk [18,19]. In retired NFL players total LDL-C was strongly correlated with the development of carotid artery plaques [20]. The changes, albeit small, observed herein are characteristically positive considering increases in RLP are associated with cardiovascular complications [21,22]. It has been documented in previous studies that exercise may have a RLP lowering effect independent of changes in diet [23,24]. LDL-III which is a sub-fraction of LDL-C concentration that is considered small & dense, is considered atherogenic and may contribute to coronary heart disease [25]. It appears that the competitive season had a negative effect on LDL sub-fractions with the observed increase in LDL-III and subsequent decrease in mean LDL size. However, over the course of a year a favorable trend was observed with an increase in mean LDL size. Both values are worth observing, but it is difficult to interpret whether the observed changes in lipoprotein profile were beneficial or harmful among the American football players in this study. Overall it appears the changes in blood lipoprotein particle numbers of collegiate athletes over season are relatively benign.

We recognize the potential limitations of the current study, including the lack of a sedentary control and small sample size. It has been reported that seasonal changes occur in blood lipids [26]. Gordon et al. [26] reported that changes observed in body mass and diet explained less than one-third of the variation reported in a longitudinal study examining cholesterol measures in men. However, that study was limited to hypercholesterolemic middle-aged men. Identification of a sedentary population of similar body composition and size as those in the current study is difficult given the unique attributes which constitute elite athletes that Division I football athletes represent. Even when compared to athletes in a lower division, Division I athletes have a lower percentage body fat, higher fat free mass, and perform better in a battery of physical tests [27] highlighting that the physicality associated with a season of American football is likely greater in Division I schools. Further, it is likely this requires an increase in physical demands over the course of a season, compared to those in lower divisions. However, we cannot rule out the possibility that seasonal changes do exist in this population. Future studies are needed to examine these changes over a period of several years.

Kirwan et al. [8] reported significant elevations in TCHL and LDL-C in fifteen (n = 15) freshmen football athletes following an 8-week strength and conditioning program. Using the standard effect size from that study to determine the minimal sample size required for significance with a desired power level of 0.80 and an alpha of 0.05 resulted in fewer participants than in the current study. Still we report no significant time effect in lipids over the course of the season, which contrasts those reported by Kirwan et al. [8]. The emphasis on resistance training with no apparent conditioning is likely responsible for the divergent findings. While weight training for 30 minutes or more per week has been associated with a significant reduction (23%) in CVD risk [28], this type of training does not seem to alter blood lipid or lipoprotein profiles [1]. Kirwan et al. [8] further suggested the increased TCHL and LDL-C observed may have been a result of overfeeding, as participants gained lean mass and fat mass while reporting caloric intake below estimated requirements. In the current study, we did not obtain dietary information, a recognized limitation. Overfeeding to achieve a desired body mass has been reported in football athletes, especially freshmen football players [29].

In summary, this is the first study to report on changes in lipids and lipoproteins associated with a season of American football. These preliminary findings, in conjunction with the work of previous investigators, demonstrates the need for continued research to identify the potential factors related to the increased risk of CVD due to dyslipidemia, particularly in those players identified at risk (i.e. linemen) as the evidence seems consistent that over several years of competitive play, the risk for the development of CVD increases. While this may not be a result of the physical activity associated with American football, other outside factors may be playing a role. Given the large numbers of participants in the sport of American football at the high school and collegiate levels, this is a growing public health concern worthy of further study.
